# Enflurane Additive for Sodium Negative Electrodes

**DOI:** 10.1021/acsami.2c06502

**Published:** 2022-08-05

**Authors:** Bhaskar Akkisetty, Konstantinos Dimogiannis, Joanne Searle, David Rogers, Graham N. Newton, Lee R. Johnson

**Affiliations:** † Nottingham Applied Materials and Interfaces Group, School of Chemistry, University of Nottingham, Nottingham NG7 2TU, U.K.; ‡ School of Chemistry, University of Nottingham, University Park, Nottingham NG7 2RD, U.K.; § The Faraday Institution, Quad One, Harwell Science and Innovation Campus, Didcot OX11 0RA, U.K.

**Keywords:** enflurane, electrolyte additive, hard carbon, sodium metal, sodium-ion battery, solid electrolyte interphase

## Abstract

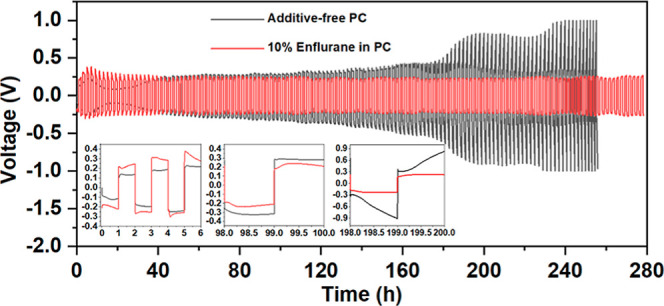

Development of sodium anodes, both hard carbon (HC) and metallic, is dependent on the discovery of electrolyte formations and additives able to stabilize the interphase and support Na^+^ transport. Halogen salt additives are known to lower the energy barrier for the Na-ion charge transfer at the interface and facilitate stable Na plating/stripping in a symmetric cell configuration. Here, a halogen-rich additive for the sodium-ion battery electrolyte, 2-chloro-1,1,2-trifluoroethyl difluoromethyl ether (enflurane), is reported. Enflurane offers a simple molecular alternative to salt-based additives. The additive is also shown to improve the cycling performance of sodium metal electrodes. Our analysis demonstrates that enflurane is preferentially reduced at the HC electrode over propylene carbonate and is incorporated into the solid electrolyte interphase (SEI). The result is a thin, halogen-rich SEI that offers better charge transport properties and stability during cycling compared to that formed in the additive-free electrolyte. Additionally, enflurane inhibits polarization of metallic sodium electrodes, and when included in HC half-cells at 10 v/v %, it improves the reversible specific capacity and stability.

## Introduction

Due to the widespread availability and low cost of sodium resources, Na-ion batteries are considered a promising alternative to the Li-ion technology, particularly in the grid and other stationary storage applications. Over the last decade, various positive electrodes (intercalation-type, oxygen, and sulfur)^1^ and negative electrodes [hard carbon (HC), phosphorus, and metallic sodium] have been reported.^2^ Of these, HC is the leading candidate in negative electrode materials and can offer capacities between ∼150 and 350 mA h g^–1^,^3−8^ while metallic sodium is preferred for next-generation systems using sulfur and oxygen.

The conventional Li-ion battery organic carbonate electrolytes are unstable when used in sodium batteries. Both the sodium metal (Na) and sodiated HC (Na_*x*_-HC) electrodes are highly reactive with the alkyl carbonate solvents, such as ethylene carbonate (EC), propylene carbonate (PC), dimethyl carbonate (DMC), and diethyl carbonate (DEC). The major decomposition products, including sodium ethylene mono and dicarbonates that form the solid electrolyte interphase (SEI), are soluble in the electrolyte solution. This is due to the weaker acidity and larger radii of Na^+^ ions compared to Li^+^, leading to instability, irreversible capacity loss, and poor cycling behavior.^9−12^ A stable and ionically conductive SEI is required for good electrochemical performance, suggesting an alternative class of electrolytes is required.

In the Li-ion battery, vinylene carbonate (VC), fluoroethylene carbonate (FEC), and sulfur containing compounds such as ethylene sulfite (ES) additives have been used to combat decomposition of the carbonate electrolytes and inhibit cell failure.^13−19^ In the Na-ion systems, VC and EC additives are unfortunately detrimental to cycling behavior, whereas research into FEC remains debatable.^5,6,8,10,12,20−22^ A recent study demonstrated that a thin passivation layer of sodium bromide (NaBr) salt lowers the energy barrier for the Na-ion charge transfer at the interface and facilitates stable Na plating/stripping in a symmetric cell configuration.^23^ Similarly, introduction of Bismuth, leading to a Na–Bi alloy electrode, reduces the interfacial tension, improves the Na-ion mobility, and suppresses the dendrite growth.^24^ An alternative is the use of solid-state electrolytes, which have been shown to reduce the flammability issues of Li metal batteries, while showing good compatibility with the Li metal electrodes.^25,26^

Chlorinated additives have been found to improve the electrochemical performance of Li metal batteries, due their desired properties such as high ionic conductivity and electrochemical stability.^27,28^ However, in large concentrations, Cl^–^ ions can induce anodic corrosion of the current collector,^29^ making the use of bulk simple Cl^–^ salts (LiClO_4_) impractical. Thus, there is a need to identify Cl^–^ electrolyte additives able to impart both stability and conductivity to the SEI at sodium metal electrodes, similar to their lithium analogues, all without the introduction of significant quantities of dissolved chloride.

Here, a halogen-rich electrolyte additive for sodium batteries, 2-chloro-1,1,2-trifluoroethyl difluoromethyl ether (enflurane), is reported. Enflurane is a non-flammable, colorless, volatile liquid at room temperature and atmospheric pressure. Its boiling point is 56.8 °C, and vapor pressure is 175 mm Hg at room temperature (20 °C). The non-flammability of enflurane is expected to be advantageous for battery applications.^30^ Enflurane offers a simple molecular alternative to salt-based additives. HC half-cells exhibited an improved reversible specific capacity during long-term cycling with 10 v/v % enflurane additive in PC compared with additive-free electrolytes. Addition of 10 v/v % enflurane to a PC electrolyte allows stable plating and stripping of metallic sodium for over 240 h. Chemical and computational analyses confirms that enflurane is preferentially reduced at the negative electrode over PC and is incorporated into the SEI, while also minimizing further decomposition of the electrolyte. The resulting SEI offers improved charge transport properties during cycling compared to that formed in the additive-free electrolyte.

## Results and Discussion

### Impact of Enflurane on Sodium Metal Cycling

**Figure 1:**
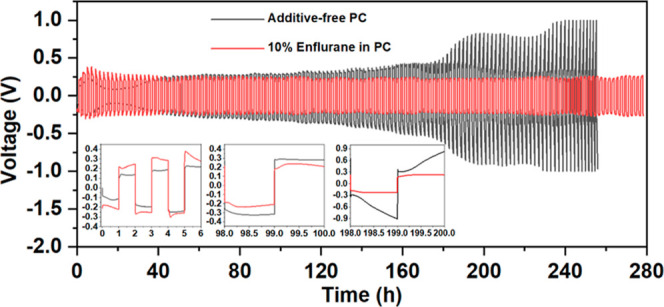
Na plating/stripping behavior in a symmetric Na–Na cell configuration, with and without 10 v/v % enflurane in 1 M NaPF_6_ PC. The current density was 0.25 mA cm^–2^ and the capacity 0.25 mA h cm^–2^, with each cycle of 2 h in duration. The insets highlight selected regions of the cycling.

The impact on sodium metal of adding 10 v/v % enflurane to PC electrolyte solutions was studied by performing cycling measurements using a symmetric Na–Na cell configuration. Na plating and stripping were performed at 0.25 mA h cm^–2^Figure 1. During the 20 h of the initial cycling, the average Na plating/stripping overpotential was ca. 100 and 250 mV for the additive-free and 10 v/v % enflurane containing PC electrolytes, respectively. The 10% enflurane additive cell showed a slight increase in polarization when compared to additive free cell but only during the initial Na plating/stripping cycles. A similar performance was also observed in previous reports with electrolyte additives such FEC, InF_3_, and polyoxometalate.^31−35^ However, after extended cycling, the stripping/plating performance of the additive-free electrolyte showed an unstable behavior, and the overpotential gradually increased to ∼900 mV by 200 h, Figure 1. After cycling for 250 h, the cell was disassembled inside the glovebox. It was observed that the separator had darkened greatly in color, indicating significant degradation. In contrast, the enflurane additive cell provided a stable Na cycling behavior, and no increase in overpotential was observed, even after 278 h. Moreover, no change in separator color was noticed, indicating a stable interphase between Na metal and the electrolyte solution. To get a better understanding of the SEI, SEM and EDX were performed on Na metal electrodes cycled with and without the additive, Figures S1 and S2. In the absence of enflurane, SEM images showed a low-quality SEI, which was more dendritic and less uniform than the SEI with enflurane in the system. Similarly, EDX showed a highly uneven distribution of degradation species, with some areas being rich in O and P degradation species due to the electrolyte decomposition. In contrast, a smoother SEI was formed when enflurane was used, and the SEI composition was homogeneous.

### HC Cycling with Enflurane Additive

**Figure 2:**
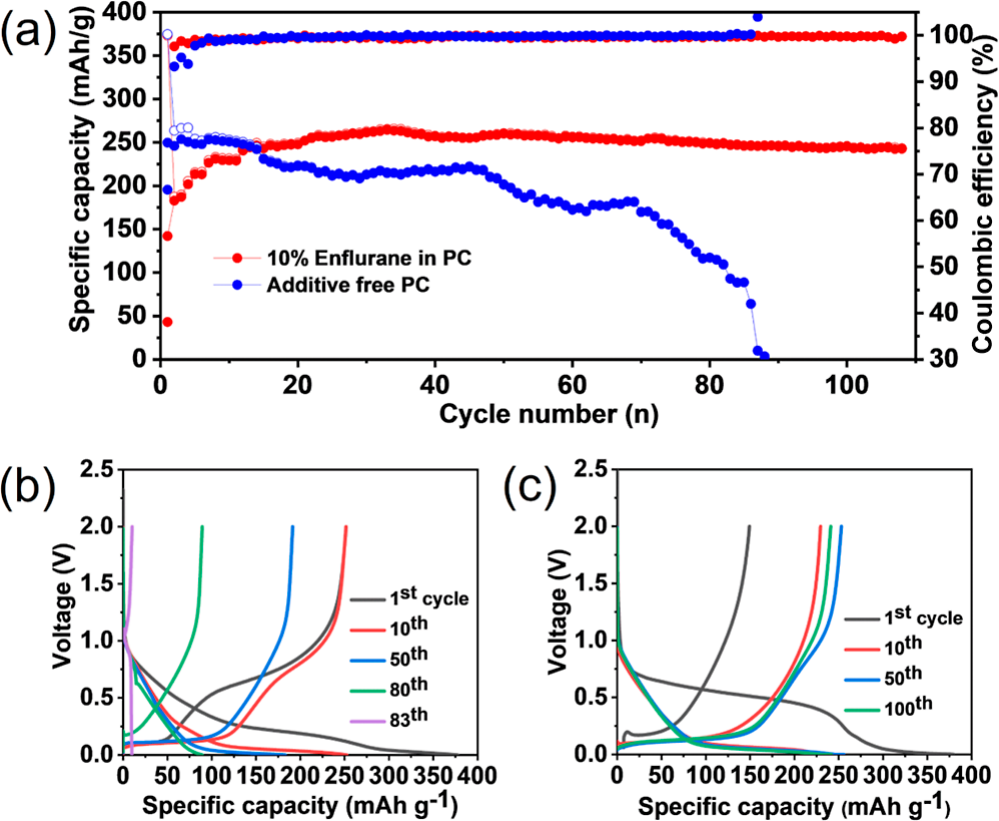
Electrochemical response of HC half-cells, with and without enflurane, in 1 M NaPF_6_ PC. (a) Comparison of the cycling performance. Charge–discharge profiles and Coulombic efficiency trends for (b) additive-free (c) enflurane containing cells. The data were obtained by applying a formation step of two cycles at 20 mA g^–1^, followed by cycling at 50 mA g^–1^ between 2 and 0 V versus Na^+^/Na.

The discharge/charge cycling response of HC electrodes with 10 v/v % enflurane and additive-free PC electrolytes are shown in Figure 2. Based on our cycling experiments with different amounts of enflurane, Figure S3, it was found 10 v/v % gives the best cycling performance and long-term stability, and this concentration was used for all further measurements. The cycling profile consisted of an initial formation step of two cycles at 20 mA g^–1^ to attain a stable SEI, followed by cycling at 50 mA g^–1^ between 2 and 0 V versus Na^+^/Na. The HC electrode cycled with enflurane showed an initial low capacity of 142 mA h g^–1^ (based on the charging capacity), due to the formation of the SEI layer during the formation step, which gradually increased up to 265 mA h g^–1^ by the end of 35 cycles, before stabilizing to a reversible capacity of 243 mA h g^–1^, Figure 2a. This represents 92% of its maximum capacity and was retained for 100 cycles. In contrast, the HC electrode with the additive-free PC electrolyte demonstrated an initially reversible specific capacity of ∼250 mA h g^–1^, which steadily decayed with cycling, until a sudden drop in capacity was observed after ca. 80 cycles, Figure 2a. The charge–discharge profiles from HC-Na cells, with and without the additive in PC electrolytes, are compared in Figure 2b,c. The first discharge profile (formation step) with additive-free PC electrolyte showed a typical cycling profile for HC, specifically, a continuous sloping region between the potentials of ∼1.0–0.1 V. This corresponds to the adsorption of Na ions at the pores and edges of the randomly oriented graphitic parallel layers. Following this was a plateau region at around 0.1 V, attributed to Na ion insertion into the nanopores and the defects in the HC.^6^ This capacity can also be partly assigned to the decomposition of the electrolyte solution to generate the SEI layer at the HC surface.^8^ In contrast, the cells containing 10 v/v % enflurane showed a unique plateau at ∼0.6 V, which we assign to decomposition of the enflurane additive to generate an interphase layer on the HC surface. The data shows that in comparison to the additive-free cell, the enflurane containing cell displayed stable cycling profiles and good capacity retention. To understand the impact of enflurane within the cell, density functional theory (DFT) was used to determine the standard reduction potentials, *E*_abs_, and molecular orbital energies of both PC and enflurane (see the Supporting Information and Figure S4 for further details). The computational analysis shows that *E*_abs_ for PC and enflurane was −0.74 and 1.57 V, respectively. In addition to this, the HOMO/LUMO energies of the additive were −9.33/–0.71 eV, compared to −8.30/–0.24 eV for PC. This suggests that enflurane should be preferentially reduced within the cell and thus incorporated into the SEI.

### Chemical Characterization of the Interphase at HC with Enflurane

**Figure 3:**
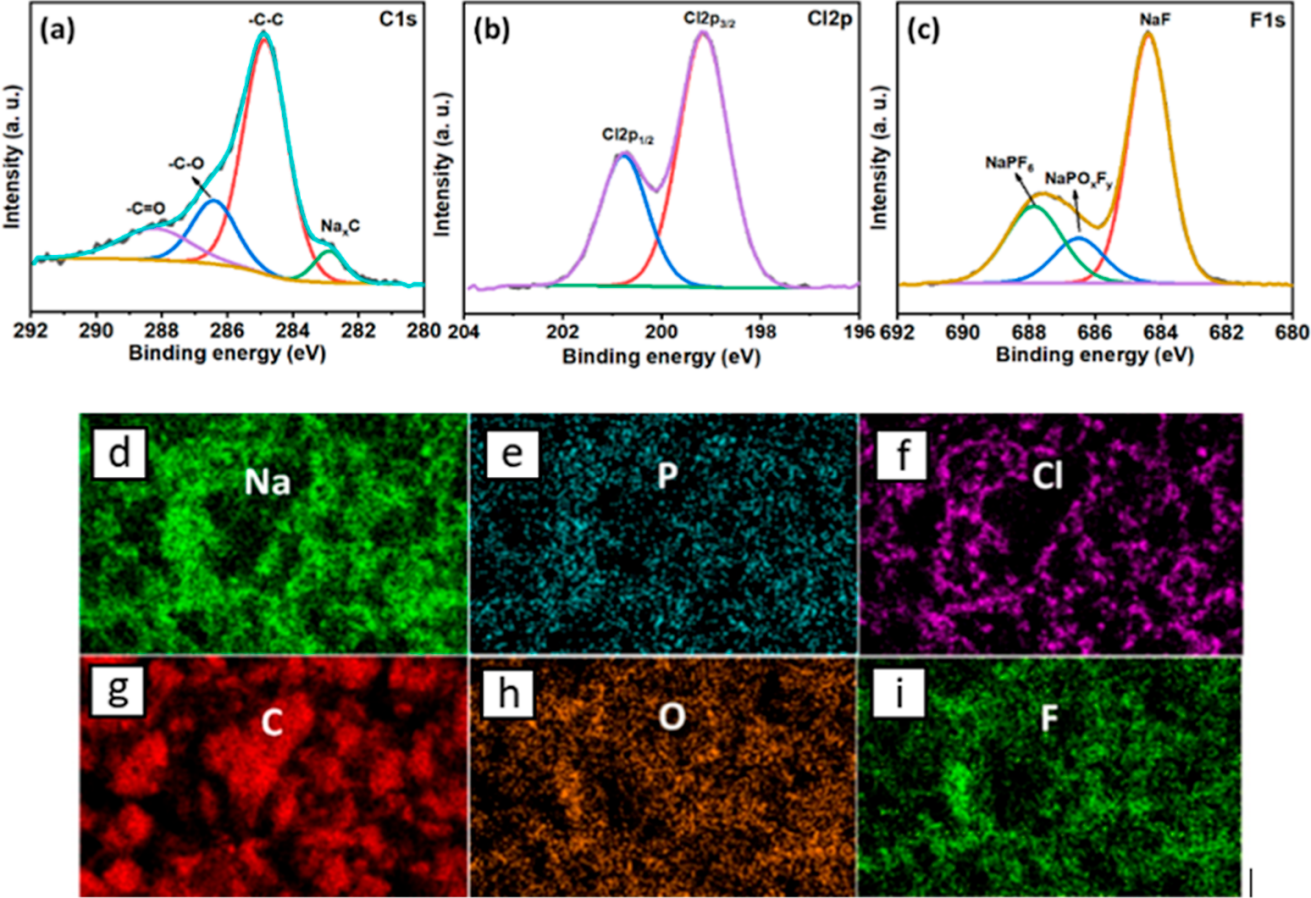
XPS (a) C 1s, (b) Cl 2p, (c) F 1s spectra, and (d–i) SEM–EDX mapping of HC after 10 cycles in a half-cell containing 10 v/v % enflurane additive in 1 M NaPF_6_ in PC at 50 mA g^–1^. A SEM image and EDS spectrum of the area is shown in Figure S6.

The interphase on the HC electrode after 10 charge–discharge cycles using 10 v/v % enflurane in the PC electrolyte was characterized by X-ray photoelectron spectroscopy (XPS) and energy-dispersive X-ray spectroscopy (EDX) elemental mapping, Figure 3. The C 1s spectra exhibited a strong peak of −C–C at 284.5 eV and weak peaks related to −C–O (286.3 eV) and C=O or O–C–O (288 eV), consistent with the formation of an SEI with a significant organic component, Figure 3a.^36−38^ The weak intensity peak at ∼283 eV corresponds to sodiated HC (Na_*x*_C). This observation confirms that the SEI is relatively thin, ca. <10 nm.^23^ The Cl 2p spectra showed a doublet corresponding to NaCl, and the F 1s spectra contained a strong peak from NaF at 684.4 eV, Figure 3b,c. Both are consistent with the decomposition of enflurane and its incorporation into the SEI at HC during cycling. A minor peak for Na_*x*_PF_*y*_ can be seen at 687.6 eV in the F 1s region, consistent with the decomposition of the electrolyte salt.^36−39^ The EDX mapping predominantly show signals corresponding to Cl, Na, and F, further confirming the presence of NaCl and NaF within the interphase, Figure 3d–i. XPS of the additive-free HC electrode showed the absence of a Na_*x*_C peak, indicating a thicker SEI, Figure S5. XPS analysis also shows a higher ratio of Na_*x*_PF_*y*_/NaPO_*x*_F_*y*_ to NaF, indicating greater amounts of electrolyte degradation, Table S1. In summary, spectroscopic analysis of the SEI at HC reveals that during charge–discharge cycling, enflurane undergoes decomposition to produce an inorganic halogen-rich interphase containing NaCl and NaF. Such an SEI is known to offer good ionic conductance and facile charge transfer to the underlying electrode.^23,40−42^

### Impedance of the Interphase at HC with Enflurane

**Figure 4:**
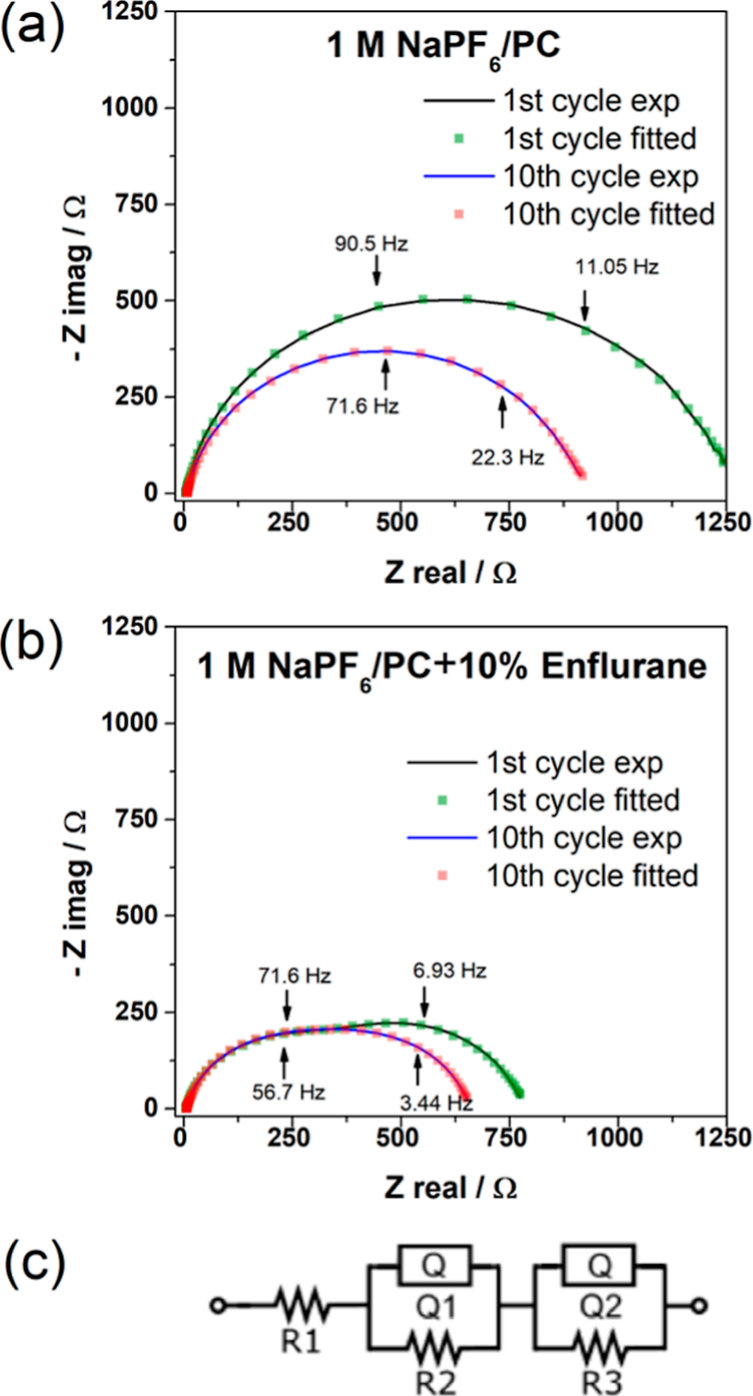
Nyquist plots obtained from HC half-cells in a three-electrode configuration after 1 and 10 cycles (a) without and (b) with 10 v/v % enflurane in 1 M NaPF_6_ PC, (c) circuit diagram used during EIS analysis. The cycling conditions were the same as those used in Figure 2. Impedance fitting data can be found in Table S2.

Electrochemical impedance spectroscopy (EIS) was used to examine the charge-transfer resistance at the SEI formed with and without enflurane in the PC electrolyte after 1 and 10 cycles, at 100% SoC (HC fully sodiated), Figure 4. The impedance of the anode in the standard electrolyte is 1300 Ω higher, suggesting a thick, resistive SEI, in which the charge-transfer process is more difficult. After 10 cycles, the carbon electrode in the enflurane containing electrolyte retains a smaller impedance. The impedance values for both systems decreased compared to the first cycle. This could be due to the opening of initially inaccessible pores in the HC material during the first cycles, which leads to an increase in the specific surface area of the carbon, thus facilitating the charge-transfer proccess.^24^ In all cases, the charge-transfer impedance was lower for cells containing enflurane.

## Conclusions

In summary, the addition of 2-chloro-1,1,2-trifluoroethyl difluoromethyl ether (enflurane) to alkyl carbonate-based electrolytes has been demonstrated to improve the performance of HC and metal electrodes within sodium batteries. An enhanced reversible capacity and a stable cycling behavior of a HC half-cell and Na symmetric cell were observed over 100 cycles when 10 v/v % enflurane additive in PC was used, compared to the additive-free electrolytes. Computational studies show that enflurane is preferentially reduced over PC and incorporated into the SEI. The result is a thinner SEI that offers enhanced passivation and stability. Enflurane imparts sodium chloride and fluoride salts to the SEI, which lowers its resistance and facilitates charge transport. We anticipate that enflurane will extend the cycle life of sodium batteries, and this will be explored in our future work.

## Supporting Information Available

Experimental methods; SEM images and EDX maps of Na metal after 250 h cycling, with and without enflurane; capacity retention and Coulombic efficiency of Na-HC cells with different amounts of enflurane; DFT-optimized structures of PC and enflurane after a 1 electron reduction; C 1s and F 1s XPS spectra of HC without enflurane; SEM image, EDX map, and spectrum of the HC electrode with enflurane; concentration of C and F species within the SEI (%) at HC, with and without enflurane, from XPS fitting; and resistance values of HC electrodes, with and without enflurane, from EIS fitting (PDF)
