# Supplementary material for: Enflurane Additive for Sodium Negative Electrodes
Source: ACS Appl Mater Interfaces. 2022 Aug 5;14(32):36551–6. doi: 10.1021/acsami.2c06502 (PMC9389525; doi:10.1021/acsami.2c06502)
Supplement: Supplementary file 1 — am2c06502_si_001.pdf [file am2c06502_si_001.pdf]

Enflurane additive for sodium negative electrodes

Supporting Information

Bhaskar Akkisetty[1], Konstantinos Dimogiannis[1], Joanne Searle[1], David Rogers[2], Graham Newton,[1,3] Lee R. Johnson[1,3*]

[1] Nottingham Applied Materials and Interfaces Group, School of Chemistry, University of Nottingham, NG7 2TU, UK

[2]School of Chemistry, University Park, University of Nottingham, Nottingham NG7 2RD, UK

[3]The Faraday Institution, Quad One, Harwell Science and Innovation Campus, Didcot, OX11 0RA, U.K.

Materials and Methods

All the reactants were of analytical grade and used without further purification. Anhydrous propylene carbonate and 2-Chloro-1,1,2-trifluoroethyl difluoromethyl ether (Enflurane) were purchased from Sigma-Aldrich. Sodium hexafluorophosphate and sodium metal cubes were purchased from Alfa-Aesar. Ultrapure water (resistivity~ 18.2 MΩ cm) collected through Millipore Advantage A10 system was used as solvent for the preparation of hard carbon (HC) electrode slurry.

Electrode preparation

HC negative electrode slurry was prepared by mixing 9:1:1 ratio of HC, acetylene black, and sodium carboxymethyl cellulose aqueous binder, respectively, with a few drops of water. The resultant slurry was cast on aluminum foil and dried at 80 °C for 12 h in a vacuum oven for removing the solvent. The casted and dried Al foils were calendared and cut into 12 mm discs. The active materials weights of HC (anode) were maintained in the range of 1 to 2 mg. All the electrodes (100 °C) and separators (300 °C) were dried in a Buchi glass vacuum oven prior to transfer into the glove box for the cell fabrication. Battery grade aluminum foil (~ 100 µm thick, 12 mm dia.) was used as the working electrode and a thin sheet of shiny sodium metal foil (~ 300 µm thick and 10 mm in dia.) served as both counter and reference electrodes. Distinct and fresh cells were fabricated every time to study each cathodic and anodic electrochemical stability windows of the electrolytes.

Characterization techniques

Scanning electron microscopy (SEM) and Energy Dispersive X-ray Spectroscopy (EDX) elemental mapping were performed on FEI Quanta200 3D FIB-SEM. An airlock transfer holder was used to transfer HC samples into the SEM vacuum chamber. SEM and EDX analysis for the Na metal electrodes was performed Zeiss EVO 10 MA SEM. X-ray photoelectron spectroscopy (XPS, Kratos LiPPS) with an AlK_{α} monochromatic X-ray light source was used for the determination of chemical composition. The electrodes were placed in the XPS spectrometer using an airtight transfer chamber to prevent any side reactions occurring with open atmosphere. Prior to ex-situ analysis of SEM and XPS, the post-cycling cells were dis-assembled in an argon filled glove-box (MBraun, O_2 and H_2O concentration ≤ 0.1 ppm and 0.1 ppm), rinsed with propylene carbonate solvent and dried in the antechamber for 30 minutes at room temperature.

Swagelok cells were employed for electrochemical measurements and were assembled inside an argon filled glove-box (MBraun, O_2 and H_2O concentration ≤ 0.1 ppm and 0.1 ppm). A Whatman glass microfiber filter paper functioned as the separator, with 1M $NaPF_6$ in PC solvent used as electrolyte. The trace level of moisture in our electrolyte was ~12 ppm, as characterized by Karl-fisher titration using CA-200 Coulometer (Mitsubishi). Galvanostatic charge-discharge measurements were performed on a battery testing unit (Biologic, BCS, BT 800) at room temperature. Electrochemical impedance spectroscopy (EIS) measurements were performed on the Biologic SP 300, within the frequency range of 200 KHz - 10 mHz, using an amplitude perturbation of 10 mV at the sodiated state after a 2 h OCV. Fitting of the EIS curves was performed using the EC-Lab software. Linear sweep voltammetry was recorded at a scan rate of 1 mV s^{-1} between 2.0 and -0.2 V in three-electrode configuration, with a carbon gas diffusion layer (GDL) as the working electrode and Na discs as the counter and reference electrodes.

Computational studies

Density functional theory (DFT) calculations were performed for molecular geometry optimization and harmonic vibrational frequency calculations, through the Q-Chem 5.0 package, by using the B3LYP hybrid functional with the 6-31+G* basis set. Reduction potentials, E_{abs}, were calculated through Equations S1 and S2, using the total DFT energy, thermal correction to enthalpy, total entropy and zero-point energy, as found in successfully converged *.out* files. HOMO/LUMO energies were also obtained from such files.

$$\Delta H_{Therm} = \Delta Total\ DFT\ energy\ + \Delta Thermal\ correction\ to\ enthalpy \qquad \text{equ. S1}$$

$$E_{abs} = \frac{(\Delta H_{Therm} - T\Delta S + \Delta ZPE)}{-nF} \qquad \text{equ. S2}$$

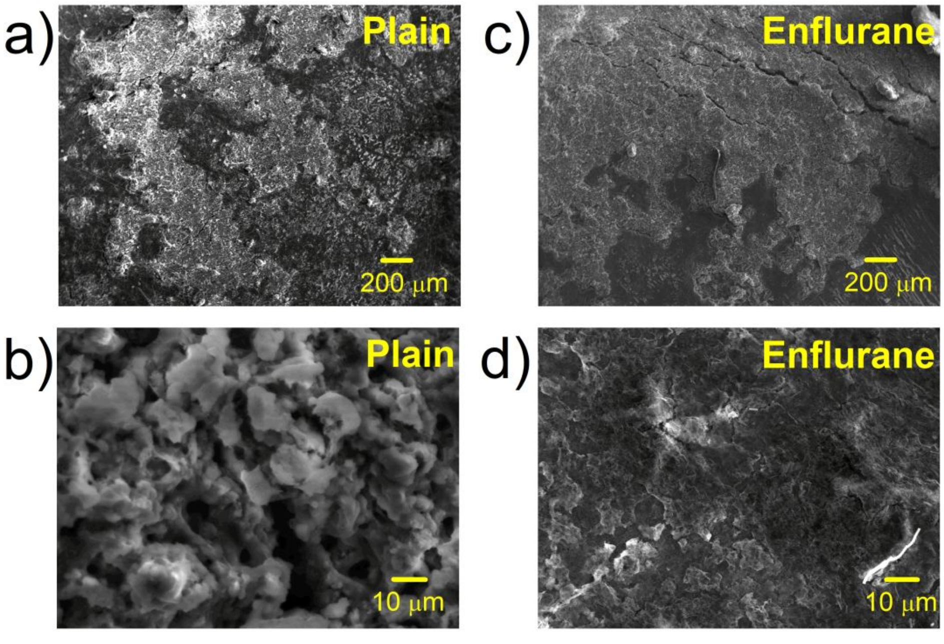

Figure S1. SEM micrographs of sodium electrodes post-cycling, (a-b) without and (c-d) with enflurane at two different magnifications. Cycling conditions are the same as used in Figure 1.

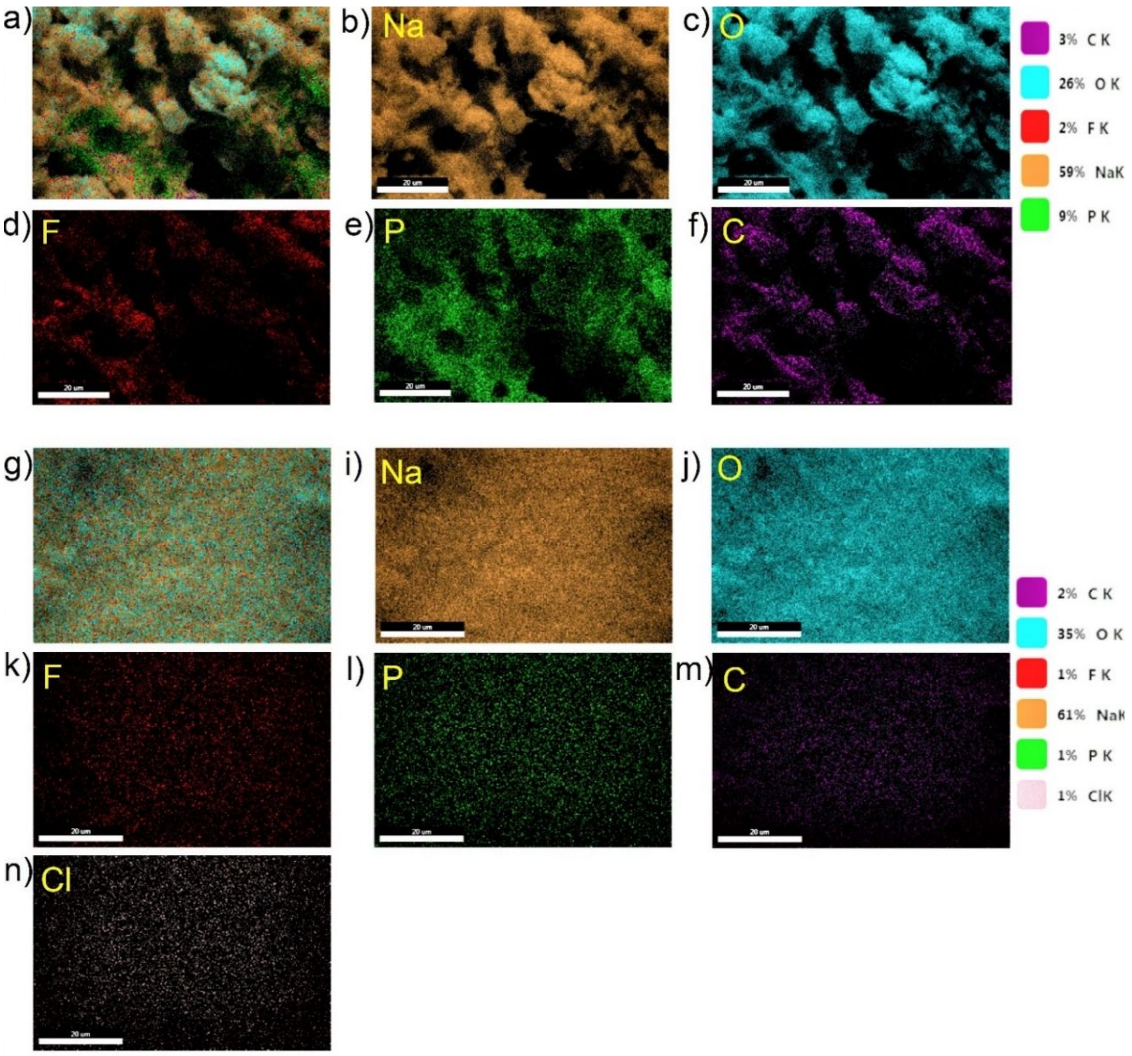

Figure S2. EDX images of the Na metal electrodes post-cycling, (a-f) without enflurane and (g-n) with enflurane. Cycling conditions are the same as used in Figure 1.

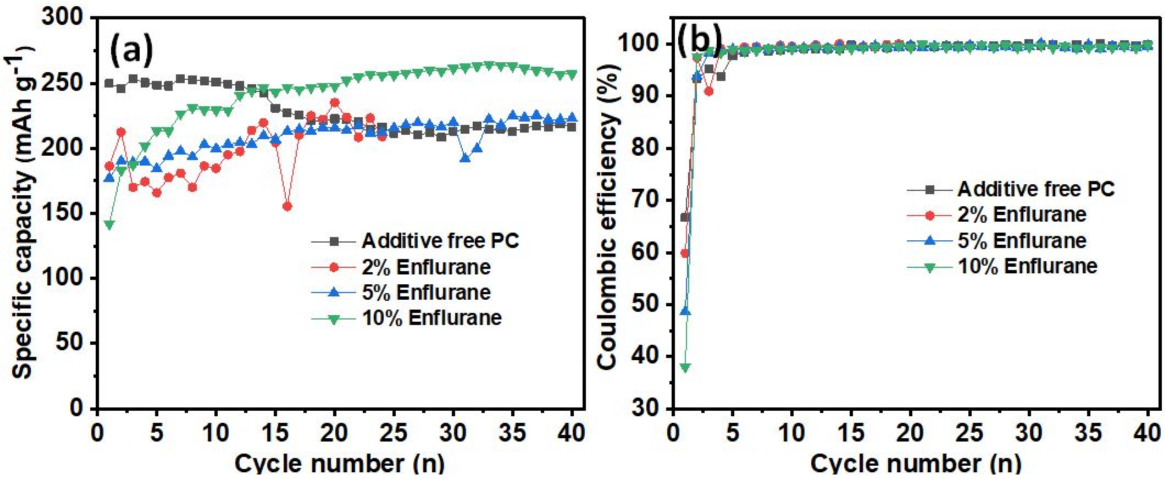

Figure S3. Comparison of cycling retention (a) and coulombic efficiency (b), for the first 40 cycles, with different amounts of enflurane in the electrolyte. The data was obtained by applying a formation step of two cycles at 20 mA g^{-1}, followed by cycling at 50 mA g^{-1} between 2 V and 0 V *vs.* Na^+/Na.

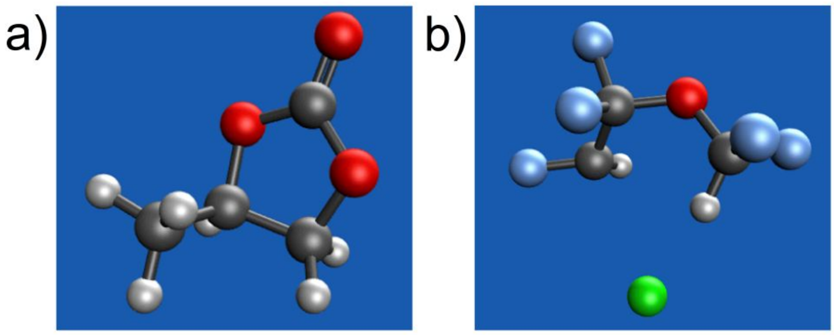

Figure S4. DFT optimized structures of (a) PC and (b) enflurane after a 1 electron reduction, displayed using IQmol.

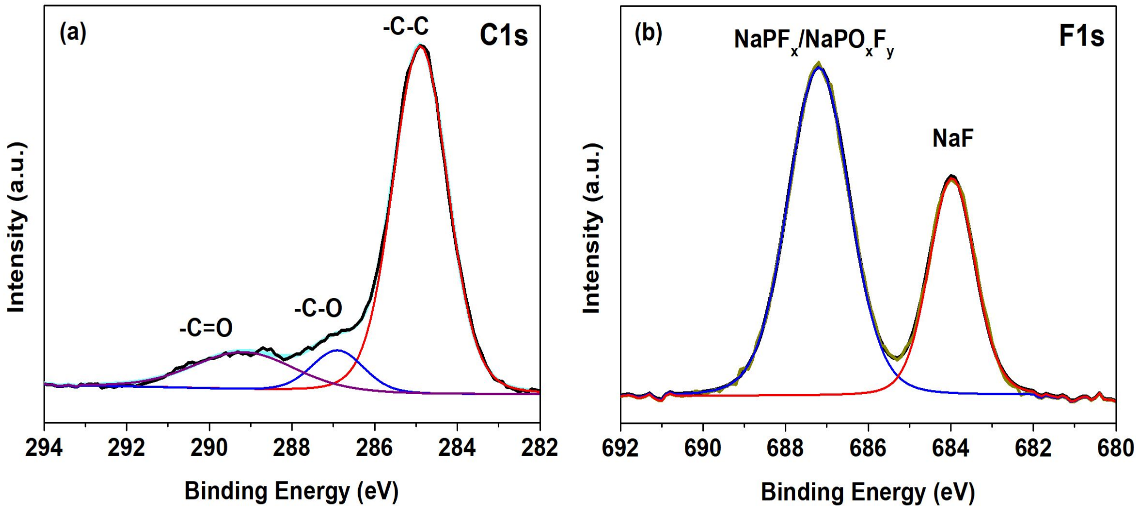

Figure S5. XPS (a) C 1s (b) F 1s spectra of HC after 10 cycles in 1 M $NaPF_6$ in PC without enflurane at 50 mA g^{-1}.

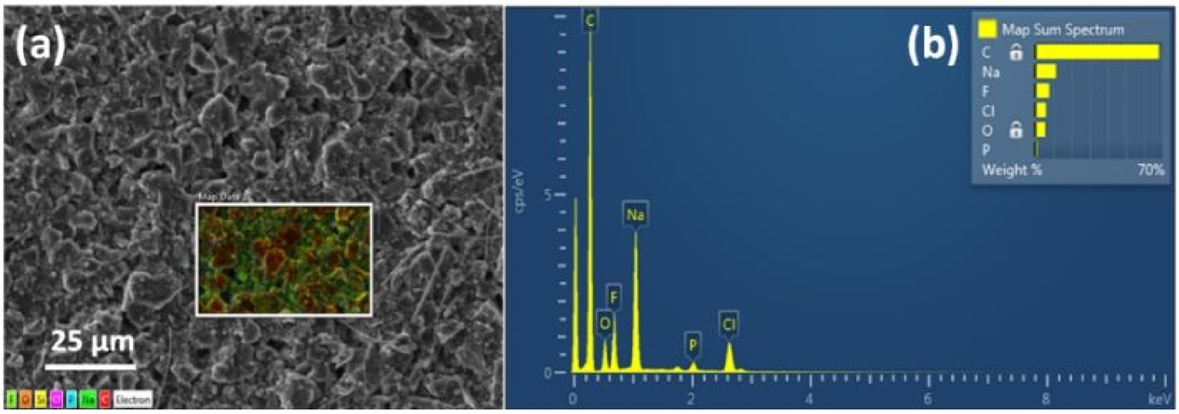

Figure S6. (a) SEM-EDX mapping and (b) spectrum of the HC electrode surface after 10 cycles in a half-cell containing 10% enflurane in 1 M $NaPF_6$ PC at 50 mA g^{-1}.

Table S1. C and F species within the SEI (%) at HC, with and without enflurane after 10 cycles at 50 mA g^{-1}.

SEI Species	Plain system (%)	Enflurane system (%)
**Na_x-HC**	0	5.86
**-C-C**	76.97	63.18
**-C-O**	11.22	17.15
**-C=O**	11.81	13.81
**NaF**	41.23	67.95
**$NaPO_xF_y$ + Na_xPF_y**	58.77	32.05

Table S2. EIS fitting data for the R and Q parameters after 1 & 10 cycles for both systems.

Circuit component value	Plain 1st sodiation	Plain 10th sodiation	Enflurane 1st sodiation	Enflurane 10th sodiation
**R1 (Ohm)**	6.316	7.223	4.452	5.48
**Q2 (µF)**	4.555	7.842	4.508	6.55
**a2**	0.706	0.605	0.803	0.730
**R2 (Ohm)**	886	889.9	426.1	480.1
**Q3 (µF)**	77.37	6.214	64.97	7.132
**a3**	0.599	0.877	0.857	0.798
**R3 (Ohm)**	345.4	220.8	297.6	167.9
